# Dibutyl 5-[(4-ethoxycarbonylphenyl)diazenyl]benzene-1,3-dicarboxylate

**DOI:** 10.1107/S1600536810022762

**Published:** 2010-06-23

**Authors:** Ying Liu, Xianxi Zhang, Zechun Xue, Jian Sheng

**Affiliations:** a College of Chemistry and Chemical Engineering, Liaocheng University, Liaocheng, 252059, People’s Republic of China

## Abstract

In the title compound, C_25_H_30_N_2_O_6_, the dihedral angle between the aromatic rings is 3.79 (1) Å and the N=N bond shows a *trans* conformation. Both butyl side chains show evidence of disorder.

## Related literature

For general background to dendrimers related to the title compound, see: Tomalia *et al.* (1990 ▶); Bosman *et al.* (1999 ▶). For a related structure, see: Wang *et al.* (2004 ▶).
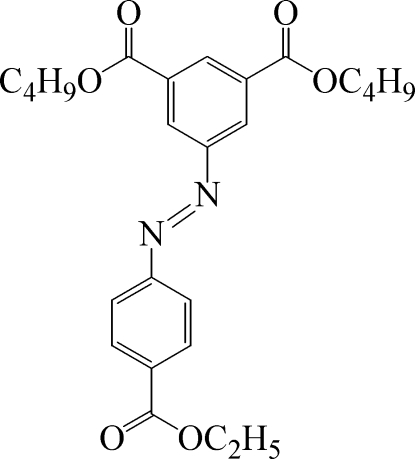


## Experimental

### 

#### Crystal data

C_25_H_30_N_2_O_6_
*M* *_r_* = 454.51
Triclinic, 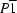

*a* = 8.675 (2) Å
*b* = 11.299 (3) Å
*c* = 13.636 (3) Å
α = 97.311 (3)°
β = 94.806 (3)°
γ = 109.793 (2)°
*V* = 1235.8 (5) Å^3^
*Z* = 2
Mo *K*α radiation
μ = 0.09 mm^−1^
*T* = 293 K
0.12 × 0.10 × 0.08 mm

#### Data collection

Bruker APEXII CCD diffractometer
Absorption correction: multi-scan (*SADABS*; Bruker, 2001 ▶) *T*_min_ = 0.990, *T*_max_ = 0.993
8643 measured reflections
4275 independent reflections
2736 reflections with *I* > 2σ(*I*)
*R*_int_ = 0.022

#### Refinement

*R*[*F* ^2^ > 2σ(*F* ^2^)] = 0.064
*wR*(*F* ^2^) = 0.218
*S* = 1.05
4275 reflections
302 parameters
13 restraints
H-atom parameters constrained
Δρ_max_ = 0.55 e Å^−3^
Δρ_min_ = −0.32 e Å^−3^

### 

Data collection: *APEX2* (Bruker, 2004 ▶); cell refinement: *SAINT-Plus* (Bruker, 2001 ▶); data reduction: *SAINT-Plus*; program(s) used to solve structure: *SHELXS97* (Sheldrick, 2008 ▶); program(s) used to refine structure: *SHELXL97* (Sheldrick, 2008 ▶); molecular graphics: *SHELXTL* (Sheldrick, 2008 ▶); software used to prepare material for publication: *SHELXTL*.

## Supplementary Material

Crystal structure: contains datablocks global, I. DOI: 10.1107/S1600536810022762/hb5477sup1.cif

Structure factors: contains datablocks I. DOI: 10.1107/S1600536810022762/hb5477Isup2.hkl

Additional supplementary materials:  crystallographic information; 3D view; checkCIF report

## Figures and Tables

**Fig. 1.**
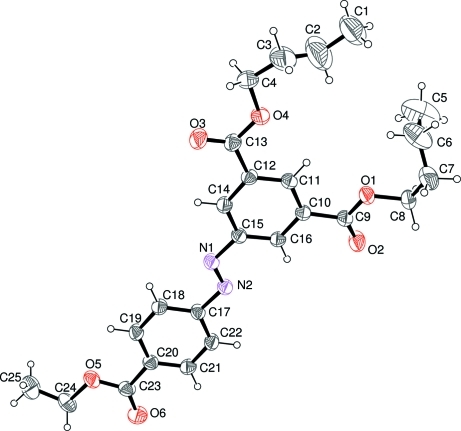
The molecular sturcture of (I) with displacement ellipsoids drawn at the 30% probability level; H atoms are given as spheres of arbitrary radius.

## supplementary crystallographic information

### Comment

Dendrimers have been the subject of intense investigation due to both interesting structural properties and promising applications in the areas of biological and material sciences (Tomalia *et al.*, 1990; Bosman *et al.*, 1999). Here, we describe the cystallization and structural characterization of the title compound.

As shown in Fig 1. the dihedral angle between the phenyl planes of the two benzene rings is 3.79 (1) Å. The mean deviations for the two phenyl planar are 0.0058 (1) and 0.0028 (1) Å, respectively. The C=O and C—O bond distances of carbonyl groups are 1.195 (3)—1.200 (3) and 1.324 (3)—1.452 (3) Å, respectively. The N=N and N—C bond distances are 1.240 (3) and 1.423 (3)—1.431 (3) Å, respectively, which are in the normal range compared to reported Dendrimer derivatives (Wang *et al.*, 2004).

### Experimental

A yellow powder of ethyl 4-((dibutyl-3',5'-biscarbonylphenyl)diazenyl)benzoate (Jinan Henghua Science & Technology Co. Ltd.) (1 mmol 0.45 g) was dissolved in 10 ml ethanol and evaporated in an open flask at room temperature. Three days later, orange blocks of (I) were obained. Anal. C_25_H_30_N_2_O_6_: C, 66.01; H, 6.60; N, 6.16%. Found: C, 65.98; H, 6.47; N, 6.05%.

### Refinement

Hydrogen atoms were placed in geometrically calculated positions (C—H 0.95 Å for aromatic and formyl, 0.99 Å for methylene and 0.98 Å for methyl) and included in the refinement in a riding motion approximation with *U*_iso_(H) = 1.2Ueq(C) [for methyl groups *U*_iso_(H) = 1.5Ueq(C)].

### Crystal data

**Table:**

C_25_H_30_N_2_O_6_	*Z* = 2
*M**_r_* = 454.51	*F*(000) = 484
Triclinic, *P*1	*D*_x_ = 1.221 Mg m^−^^3^
Hall symbol: -P 1	Mo *K*α radiation, λ = 0.71073 Å
*a* = 8.675 (2) Å	Cell parameters from 2660 reflections
*b* = 11.299 (3) Å	θ = 2.5–27.1°
*c* = 13.636 (3) Å	µ = 0.09 mm^−^^1^
α = 97.311 (3)°	*T* = 293 K
β = 94.806 (3)°	Block, yellow
γ = 109.793 (2)°	0.12 × 0.10 × 0.08 mm
*V* = 1235.8 (5) Å^3^	

### Data collection

**Table:**

Bruker APEXII CCD diffractometer	4275 independent reflections
Radiation source: fine-focus sealed tube	2736 reflections with *I* > 2σ(*I*)
graphite	*R*_int_ = 0.022
φ and ω scans	θ_max_ = 25.0°, θ_min_ = 2.5°
Absorption correction: multi-scan (*SADABS*; Bruker, 2001)	*h* = −10→10
*T*_min_ = 0.990, *T*_max_ = 0.993	*k* = −13→13
8643 measured reflections	*l* = −15→16

### Refinement

**Table:**

Refinement on *F*^2^	Primary atom site location: structure-invariant direct methods
Least-squares matrix: full	Secondary atom site location: difference Fourier map
*R*[*F*^2^ > 2σ(*F*^2^)] = 0.064	Hydrogen site location: inferred from neighbouring sites
*wR*(*F*^2^) = 0.218	H-atom parameters constrained
*S* = 1.05	*w* = 1/[σ^2^(*F*_o_^2^) + (0.1149*P*)^2^ + 0.3064*P*] where *P* = (*F*_o_^2^ + 2*F*_c_^2^)/3
4275 reflections	(Δ/σ)_max_ < 0.001
302 parameters	Δρ_max_ = 0.55 e Å^−^^3^
13 restraints	Δρ_min_ = −0.32 e Å^−^^3^

### Special details

**Table:**

Geometry. All e.s.d.'s (except the e.s.d. in the dihedral angle between two l.s. planes) are estimated using the full covariance matrix. The cell e.s.d.'s are taken into account individually in the estimation of e.s.d.'s in distances, angles and torsion angles; correlations between e.s.d.'s in cell parameters are only used when they are defined by crystal symmetry. An approximate (isotropic) treatment of cell e.s.d.'s is used for estimating e.s.d.'s involving l.s. planes.
Refinement. Refinement of *F*^2^ against ALL reflections. The weighted *R*-factor *wR* and goodness of fit *S* are based on *F*^2^, conventional *R*-factors *R* are based on *F*, with *F* set to zero for negative *F*^2^. The threshold expression of *F*^2^ > σ(*F*^2^) is used only for calculating *R*-factors(gt) *etc*. and is not relevant to the choice of reflections for refinement. *R*-factors based on *F*^2^ are statistically about twice as large as those based on *F*, and *R*- factors based on ALL data will be even larger.

### Fractional atomic coordinates and isotropic or equivalent isotropic displacement parameters (Å^2^)

**Table:**

	*x*	*y*	*z*	*U*_iso_*/*U*_eq_	
O1	1.0434 (3)	0.2950 (2)	0.48258 (15)	0.0725 (6)	
O2	0.8502 (3)	0.3718 (2)	0.52977 (16)	0.0932 (8)	
O3	0.7562 (3)	−0.0204 (2)	0.05822 (16)	0.0871 (7)	
O4	0.9356 (3)	−0.0106 (2)	0.18814 (15)	0.0810 (7)	
O5	−0.2310 (2)	0.3824 (2)	0.02977 (15)	0.0714 (6)	
O6	−0.1835 (3)	0.5354 (2)	0.1600 (2)	0.0974 (8)	
N1	0.4477 (3)	0.2563 (2)	0.19969 (16)	0.0588 (6)	
N2	0.4018 (3)	0.3269 (2)	0.25818 (16)	0.0577 (6)	
C1	1.3390 (8)	−0.0964 (7)	0.3283 (5)	0.173 (3)	
H1A	1.3681	−0.1621	0.2913	0.260*	
H1B	1.3211	−0.1154	0.3940	0.260*	
H1C	1.4271	−0.0159	0.3333	0.260*	
C2	1.1938 (10)	−0.0905 (10)	0.2790 (6)	0.225 (4)	
H2A	1.1052	−0.1568	0.3004	0.270*	
H2B	1.1878	−0.0105	0.3104	0.270*	
C3	1.1450 (9)	−0.0977 (7)	0.1825 (5)	0.189 (3)	
H3A	1.1593	−0.1744	0.1507	0.227*	
H3B	1.2301	−0.0275	0.1625	0.227*	
C4	0.9940 (5)	−0.0991 (4)	0.1300 (3)	0.0905 (11)	
H4A	0.9116	−0.1843	0.1198	0.109*	
H4B	1.0129	−0.0745	0.0652	0.109*	
C5	1.2579 (9)	0.1314 (8)	0.5848 (7)	0.207 (4)	
H5A	1.2809	0.1655	0.6548	0.311*	
H5B	1.3017	0.0643	0.5720	0.311*	
H5C	1.1405	0.0980	0.5646	0.311*	
C6	1.3321 (7)	0.2288 (7)	0.5303 (5)	0.149 (2)	
H6A	1.2823	0.2014	0.4611	0.179*	
H6B	1.4485	0.2407	0.5327	0.179*	
C7	1.3166 (5)	0.3532 (4)	0.5670 (4)	0.1102 (14)	
H7A	1.3649	0.4117	0.5223	0.132*	
H7B	1.3831	0.3866	0.6319	0.132*	
C8	1.1471 (4)	0.3556 (3)	0.5769 (2)	0.0842 (10)	
H8A	1.1025	0.3102	0.6298	0.101*	
H8B	1.1516	0.4429	0.5931	0.101*	
C9	0.8992 (4)	0.3119 (3)	0.4689 (2)	0.0642 (8)	
C10	0.8072 (3)	0.2505 (2)	0.36752 (19)	0.0545 (7)	
C11	0.8557 (3)	0.1654 (2)	0.3055 (2)	0.0552 (7)	
H11	0.9463	0.1446	0.3274	0.066*	
C12	0.7685 (3)	0.1118 (2)	0.21106 (19)	0.0527 (6)	
C13	0.8165 (3)	0.0201 (3)	0.1433 (2)	0.0615 (7)	
C14	0.6346 (3)	0.1444 (2)	0.1786 (2)	0.0569 (7)	
H14	0.5769	0.1095	0.1150	0.068*	
C15	0.5867 (3)	0.2286 (2)	0.24046 (19)	0.0523 (6)	
C16	0.6712 (3)	0.2805 (2)	0.33568 (19)	0.0532 (6)	
H16	0.6367	0.3351	0.3779	0.064*	
C17	0.2637 (3)	0.3539 (2)	0.21661 (19)	0.0523 (6)	
C18	0.1725 (3)	0.2944 (3)	0.1236 (2)	0.0603 (7)	
H18	0.2007	0.2334	0.0842	0.072*	
C19	0.0408 (3)	0.3268 (3)	0.0906 (2)	0.0608 (7)	
H19	−0.0207	0.2868	0.0287	0.073*	
C20	−0.0022 (3)	0.4184 (2)	0.1480 (2)	0.0538 (6)	
C21	0.0888 (3)	0.4765 (3)	0.2404 (2)	0.0631 (7)	
H21	0.0611	0.5380	0.2796	0.076*	
C22	0.2203 (3)	0.4437 (3)	0.2744 (2)	0.0623 (7)	
H22	0.2802	0.4824	0.3369	0.075*	
C23	−0.1457 (4)	0.4536 (3)	0.1153 (2)	0.0629 (7)	
C24	−0.3775 (4)	0.4066 (4)	−0.0072 (3)	0.0835 (10)	
H24A	−0.3473	0.4937	−0.0197	0.100*	
H24B	−0.4538	0.3948	0.0419	0.100*	
C25	−0.4555 (5)	0.3171 (5)	−0.0998 (4)	0.1176 (15)	
H25A	−0.3772	0.3263	−0.1467	0.176*	
H25B	−0.5491	0.3349	−0.1276	0.176*	
H25C	−0.4913	0.2314	−0.0860	0.176*	

### Atomic displacement parameters (Å^2^)

**Table:**

	*U*^11^	*U*^22^	*U*^33^	*U*^12^	*U*^13^	*U*^23^
O1	0.0697 (13)	0.0861 (14)	0.0657 (12)	0.0446 (11)	−0.0120 (10)	−0.0074 (10)
O2	0.0977 (17)	0.1285 (19)	0.0641 (13)	0.0739 (16)	−0.0125 (12)	−0.0253 (13)
O3	0.0918 (16)	0.1085 (17)	0.0657 (14)	0.0566 (14)	−0.0027 (12)	−0.0195 (12)
O4	0.0927 (16)	0.0967 (15)	0.0693 (13)	0.0649 (13)	−0.0007 (11)	−0.0106 (11)
O5	0.0609 (12)	0.0868 (14)	0.0751 (14)	0.0419 (11)	−0.0039 (10)	0.0088 (11)
O6	0.0949 (17)	0.1010 (17)	0.1112 (19)	0.0690 (15)	−0.0067 (14)	−0.0114 (14)
N1	0.0510 (13)	0.0669 (14)	0.0607 (14)	0.0285 (11)	0.0006 (10)	0.0014 (11)
N2	0.0523 (13)	0.0640 (13)	0.0594 (13)	0.0284 (11)	0.0014 (10)	0.0017 (10)
C1	0.140 (5)	0.215 (7)	0.181 (6)	0.091 (5)	−0.021 (4)	0.043 (5)
C2	0.186 (7)	0.343 (12)	0.185 (7)	0.177 (8)	−0.038 (6)	−0.012 (7)
C3	0.216 (6)	0.243 (6)	0.161 (5)	0.188 (5)	−0.031 (4)	−0.049 (4)
C4	0.099 (3)	0.097 (2)	0.091 (2)	0.065 (2)	0.0094 (19)	−0.0101 (18)
C5	0.173 (6)	0.213 (7)	0.302 (10)	0.105 (6)	0.090 (7)	0.137 (8)
C6	0.108 (4)	0.195 (6)	0.185 (6)	0.092 (4)	0.028 (4)	0.065 (5)
C7	0.080 (3)	0.120 (3)	0.124 (3)	0.036 (2)	−0.021 (2)	0.019 (3)
C8	0.084 (2)	0.092 (2)	0.073 (2)	0.0434 (19)	−0.0254 (17)	−0.0106 (17)
C9	0.0676 (18)	0.0685 (17)	0.0615 (17)	0.0375 (15)	−0.0027 (14)	−0.0014 (13)
C10	0.0532 (15)	0.0577 (15)	0.0550 (15)	0.0260 (12)	0.0036 (12)	0.0025 (12)
C11	0.0516 (15)	0.0594 (15)	0.0595 (16)	0.0272 (12)	0.0054 (12)	0.0073 (12)
C12	0.0508 (15)	0.0538 (14)	0.0560 (15)	0.0226 (12)	0.0106 (12)	0.0036 (11)
C13	0.0589 (17)	0.0664 (17)	0.0595 (18)	0.0265 (14)	0.0085 (14)	−0.0010 (13)
C14	0.0537 (15)	0.0601 (15)	0.0551 (15)	0.0213 (13)	0.0033 (12)	0.0027 (12)
C15	0.0460 (14)	0.0568 (14)	0.0560 (15)	0.0224 (12)	0.0043 (12)	0.0067 (11)
C16	0.0524 (15)	0.0555 (14)	0.0548 (15)	0.0259 (12)	0.0070 (12)	0.0011 (11)
C17	0.0455 (14)	0.0607 (15)	0.0524 (14)	0.0227 (12)	0.0033 (11)	0.0066 (11)
C18	0.0578 (16)	0.0670 (16)	0.0602 (16)	0.0338 (14)	0.0028 (13)	−0.0046 (13)
C19	0.0548 (16)	0.0701 (17)	0.0575 (16)	0.0295 (13)	−0.0034 (12)	−0.0038 (13)
C20	0.0486 (14)	0.0554 (14)	0.0606 (16)	0.0229 (12)	0.0065 (12)	0.0089 (12)
C21	0.0654 (18)	0.0652 (16)	0.0642 (17)	0.0353 (14)	0.0068 (14)	−0.0034 (13)
C22	0.0608 (17)	0.0711 (17)	0.0546 (16)	0.0312 (14)	−0.0024 (13)	−0.0064 (13)
C23	0.0593 (17)	0.0638 (16)	0.0738 (19)	0.0322 (14)	0.0088 (15)	0.0110 (14)
C24	0.0625 (19)	0.109 (3)	0.094 (2)	0.0478 (19)	−0.0003 (17)	0.027 (2)
C25	0.081 (3)	0.141 (4)	0.125 (3)	0.045 (3)	−0.026 (2)	0.012 (3)

### Geometric parameters (Å, °)

**Table:**

O1—C9	1.329 (3)	C7—H7A	0.9700
O1—C8	1.448 (3)	C7—H7B	0.9700
O2—C9	1.200 (3)	C8—H8A	0.9700
O3—C13	1.196 (3)	C8—H8B	0.9700
O4—C13	1.324 (3)	C9—C10	1.492 (4)
O4—C4	1.447 (3)	C10—C16	1.385 (3)
O5—C23	1.330 (3)	C10—C11	1.394 (3)
O5—C24	1.452 (3)	C11—C12	1.388 (4)
O6—C23	1.195 (3)	C11—H11	0.9300
N1—N2	1.240 (3)	C12—C14	1.388 (4)
N1—C15	1.431 (3)	C12—C13	1.488 (3)
N2—C17	1.423 (3)	C14—C15	1.383 (3)
C1—C2	1.403 (7)	C14—H14	0.9300
C1—H1A	0.9600	C15—C16	1.386 (4)
C1—H1B	0.9600	C16—H16	0.9300
C1—H1C	0.9600	C17—C22	1.378 (3)
C2—C3	1.331 (7)	C17—C18	1.393 (4)
C2—H2A	0.9700	C18—C19	1.372 (4)
C2—H2B	0.9700	C18—H18	0.9300
C3—C4	1.434 (7)	C19—C20	1.388 (3)
C3—H3A	0.9700	C19—H19	0.9300
C3—H3B	0.9700	C20—C21	1.384 (4)
C4—H4A	0.9700	C20—C23	1.481 (4)
C4—H4B	0.9700	C21—C22	1.377 (4)
C5—C6	1.409 (8)	C21—H21	0.9300
C5—H5A	0.9600	C22—H22	0.9300
C5—H5B	0.9600	C24—C25	1.465 (5)
C5—H5C	0.9600	C24—H24A	0.9700
C6—C7	1.485 (7)	C24—H24B	0.9700
C6—H6A	0.9700	C25—H25A	0.9600
C6—H6B	0.9700	C25—H25B	0.9600
C7—C8	1.496 (5)	C25—H25C	0.9600
			
C9—O1—C8	116.9 (2)	O1—C9—C10	111.9 (2)
C13—O4—C4	117.4 (2)	C16—C10—C11	120.3 (2)
C23—O5—C24	116.5 (2)	C16—C10—C9	118.2 (2)
N2—N1—C15	114.7 (2)	C11—C10—C9	121.5 (2)
N1—N2—C17	114.1 (2)	C12—C11—C10	119.8 (2)
C2—C1—H1A	109.5	C12—C11—H11	120.1
C2—C1—H1B	109.5	C10—C11—H11	120.1
H1A—C1—H1B	109.5	C14—C12—C11	119.7 (2)
C2—C1—H1C	109.5	C14—C12—C13	119.1 (2)
H1A—C1—H1C	109.5	C11—C12—C13	121.1 (2)
H1B—C1—H1C	109.5	O3—C13—O4	123.8 (2)
C3—C2—C1	131.1 (7)	O3—C13—C12	124.4 (3)
C3—C2—H2A	104.5	O4—C13—C12	111.9 (2)
C1—C2—H2A	104.5	C15—C14—C12	120.2 (2)
C3—C2—H2B	104.5	C15—C14—H14	119.9
C1—C2—H2B	104.5	C12—C14—H14	119.9
H2A—C2—H2B	105.6	C14—C15—C16	120.4 (2)
C2—C3—C4	132.1 (6)	C14—C15—N1	115.8 (2)
C2—C3—H3A	104.2	C16—C15—N1	123.8 (2)
C4—C3—H3A	104.2	C10—C16—C15	119.6 (2)
C2—C3—H3B	104.2	C10—C16—H16	120.2
C4—C3—H3B	104.2	C15—C16—H16	120.2
H3A—C3—H3B	105.5	C22—C17—C18	119.8 (2)
C3—C4—O4	108.9 (3)	C22—C17—N2	116.3 (2)
C3—C4—H4A	109.9	C18—C17—N2	123.9 (2)
O4—C4—H4A	109.9	C19—C18—C17	119.3 (2)
C3—C4—H4B	109.9	C19—C18—H18	120.3
O4—C4—H4B	109.9	C17—C18—H18	120.3
H4A—C4—H4B	108.3	C18—C19—C20	121.1 (2)
C6—C5—H5A	109.5	C18—C19—H19	119.4
C6—C5—H5B	109.5	C20—C19—H19	119.4
H5A—C5—H5B	109.5	C21—C20—C19	119.1 (2)
C6—C5—H5C	109.5	C21—C20—C23	118.5 (2)
H5A—C5—H5C	109.5	C19—C20—C23	122.4 (2)
H5B—C5—H5C	109.5	C22—C21—C20	120.2 (2)
C5—C6—C7	114.3 (6)	C22—C21—H21	119.9
C5—C6—H6A	108.7	C20—C21—H21	119.9
C7—C6—H6A	108.7	C21—C22—C17	120.5 (2)
C5—C6—H6B	108.7	C21—C22—H22	119.7
C7—C6—H6B	108.7	C17—C22—H22	119.7
H6A—C6—H6B	107.6	O6—C23—O5	123.0 (3)
C6—C7—C8	118.0 (4)	O6—C23—C20	124.8 (3)
C6—C7—H7A	107.8	O5—C23—C20	112.2 (2)
C8—C7—H7A	107.8	O5—C24—C25	108.2 (3)
C6—C7—H7B	107.8	O5—C24—H24A	110.1
C8—C7—H7B	107.8	C25—C24—H24A	110.1
H7A—C7—H7B	107.2	O5—C24—H24B	110.1
O1—C8—C7	107.9 (3)	C25—C24—H24B	110.1
O1—C8—H8A	110.1	H24A—C24—H24B	108.4
C7—C8—H8A	110.1	C24—C25—H25A	109.5
O1—C8—H8B	110.1	C24—C25—H25B	109.5
C7—C8—H8B	110.1	H25A—C25—H25B	109.5
H8A—C8—H8B	108.4	C24—C25—H25C	109.5
O2—C9—O1	124.2 (3)	H25A—C25—H25C	109.5
O2—C9—C10	123.8 (3)	H25B—C25—H25C	109.5
			
C15—N1—N2—C17	179.9 (2)	C12—C14—C15—N1	179.7 (2)
C1—C2—C3—C4	175.9 (8)	N2—N1—C15—C14	176.5 (2)
C2—C3—C4—O4	39.1 (12)	N2—N1—C15—C16	−3.3 (4)
C13—O4—C4—C3	166.9 (4)	C11—C10—C16—C15	−2.1 (4)
C5—C6—C7—C8	−54.1 (7)	C9—C10—C16—C15	177.7 (2)
C9—O1—C8—C7	−165.9 (3)	C14—C15—C16—C10	1.9 (4)
C6—C7—C8—O1	−52.5 (5)	N1—C15—C16—C10	−178.3 (2)
C8—O1—C9—O2	−1.0 (5)	N1—N2—C17—C22	−172.5 (2)
C8—O1—C9—C10	177.2 (3)	N1—N2—C17—C18	8.1 (4)
O2—C9—C10—C16	10.6 (5)	C22—C17—C18—C19	0.3 (4)
O1—C9—C10—C16	−167.7 (2)	N2—C17—C18—C19	179.6 (2)
O2—C9—C10—C11	−169.7 (3)	C17—C18—C19—C20	0.5 (4)
O1—C9—C10—C11	12.1 (4)	C18—C19—C20—C21	−0.7 (4)
C16—C10—C11—C12	0.8 (4)	C18—C19—C20—C23	−178.8 (3)
C9—C10—C11—C12	−179.0 (3)	C19—C20—C21—C22	0.1 (4)
C10—C11—C12—C14	0.7 (4)	C23—C20—C21—C22	178.2 (3)
C10—C11—C12—C13	−179.7 (2)	C20—C21—C22—C17	0.7 (5)
C4—O4—C13—O3	−0.4 (5)	C18—C17—C22—C21	−0.9 (4)
C4—O4—C13—C12	−179.8 (3)	N2—C17—C22—C21	179.7 (3)
C14—C12—C13—O3	6.7 (4)	C24—O5—C23—O6	−1.3 (5)
C11—C12—C13—O3	−173.0 (3)	C24—O5—C23—C20	178.1 (2)
C14—C12—C13—O4	−174.0 (2)	C21—C20—C23—O6	5.3 (5)
C11—C12—C13—O4	6.4 (4)	C19—C20—C23—O6	−176.7 (3)
C11—C12—C14—C15	−0.9 (4)	C21—C20—C23—O5	−174.1 (2)
C13—C12—C14—C15	179.5 (2)	C19—C20—C23—O5	3.9 (4)
C12—C14—C15—C16	−0.5 (4)	C23—O5—C24—C25	−178.6 (3)
